# Desialylation and sialidase secretion from monocytes and macrophages upon LPS activation

**DOI:** 10.3389/fimmu.2026.1750968

**Published:** 2026-04-13

**Authors:** Yu Zhao, Majdi Aljohani, Morgan Pychowycz, Xue-Long Sun

**Affiliations:** Department of Chemistry, Chemical and Biomedical Engineering and Center for Gene Regulation of Health and Disease (GRHD), Cleveland State University, Cleveland, OH, United States

**Keywords:** desialylation, LPS, sialic acid, sialidase, THP-1 macrophages, THP-1 monocytes

## Abstract

**Introduction:**

The cell surface expresses a dense layer of glycans often terminated with sialic acids (Sias), known as sialylation, which represents different cellular statuses. Meanwhile, the removal of Sias from the glycans catalyzed by sialidase (desialylation) is also involved in many biological processes, such as inflammatory responses. Previous studies demonstrated that desialylation is involved in LPS/TLR4 signaling pathway and pro-inflammatory cytokine production. In this report, we systematically profiled desialylation and sialidase activity, expression and secretion from monocytes and macrophages upon LPS activation.

**Methods:**

THP-1 monocytes and THP-1 macrophages were activated by LPS (100 ng/mL) for 24 hours. The cellular Sia changes (or desialylation) was quantified by measuring the total Sia level in cell lysates by LC-MS/MS method. The cell surface desialylation was confirmed by detecting the Gal exposure (after desialylation) of the cells by flow cytometry with PNA lectin staining. Total cellular desialylation was confirmed by lectin blot for both THP-1 monocytes and THP-1 macrophages. The sialidase activity in THP-1 monocytes and THP-1 macrophages and their culture media were determined by using the exogenous sialidase substrate 4-MU-Neu5Ac and ganglioside GM3, respectively. Neu1 and Neu3 sialidase levels in THP-1 monocytes and THP-1 were determined by western blot. Neu1 and Neu3 sialidase levels in in the supernatant of THP-1 monocytes and THP-1 macrophages were determined by ELISA.

**Results:**

The total Sia level reduced significantly in both THP-1 monocytes and THP-1 macrophages upon LPS activation. Meanwhile, sialidase expression and activity increased significantly in THP-1 monocytes and THP-1 macrophages upon LPS activation. Surprisingly, sialidase activities also significantly increased in the cell culture media of both THP-1 monocytes and THP-1 macrophages, indicating sialidase secretion from THP-1 monocytes and THP-1 macrophages upon LPS activation. In comparison, THP-1 macrophages are highly desialylated than THP-1 monocytes upon LPS activation.

**Conclusion:**

LPS activation causes significant desialylation of both THP-1 monocytes and THP-1 macrophages. Desialylation is consistent with enhanced expression and activity of Neu1 and Neu3 sialidase and their secretion from both THP-1 monocytes and THP-1 macrophages upon LPS activation.

## Introduction

Sialic acids (Sias) are acidic nine carbon monosaccharides commonly located at the terminal of glycans of both glycoproteins and glycolipids, known as sialylation (1). Sias are involved in many physiological and pathological processes, including cell adhesion, migration, inflammation, tumor progression and metastasis (2). Meanwhile, the removal of Sias from the glycans catalyzed by sialidase (desialylation) is also involved in many biological processes through changing protein conformation and assembling and unknown mechanisms (3). In mammalian cells, there are four kinds of sialidase, which are Neu1, Neu2, Neu3, and Neu4, respectively (4, 5). Neu1 is the lysosomal sialidase, which degrades sialoglycoconjugates in the lysosome (6–8); Neu2 is the cytosolic sialidase that is involved in desialylation of sialoglycoconjugates in the cytosol (9, 10); Neu3 is located at the membrane and specific for gangliosides (11); Neu4 is found on the outer membrane of mitochondria, which could work on a bunch of sialoglycoconjugates including glycoproteins, gangliosides, and oligosaccharides (12). These sialidases are involved in both physiological and pathological pathways.

In addition to specific cellular location, lysosomal sialidase Neu1 could move to the cell surface upon cell activation in several cell types, such as macrophages (13), T lymphocytes (14), and myofibroblasts (15), where it could cause the desialylation of cell surface receptors and their activation, like Toll-like receptor 4 (TLR4). TLR4 serves as a receptor for lipopolysaccharide (LPS) in monocytes and macrophages (16). Dysregulation of TLR4 activation by LPS is responsible for chronic and acute inflammatory disorders that often cause life-threatening diseases like sepsis that still lacks specific pharmacological treatment (17). Previous studies confirmed that Neu1 is involved in LPS/TLR4 signaling pathway and pro-inflammatory cytokine production (18–20). Further, macrophages could release sialidases upon activation. LPS-activated microglia releases Neu1 sialidase, which stimulates microglial phagocytosis and sensitizes neurons to glutamate (21). Microglia also secrete Neu3 associated with extracellular vesicles, which is involved in remodeling of the glycocalyx leading to aberrant network-level activity of neurons, with implications in neuroinflammatory diseases such as Parkinson’s disease and Alzheimer’s disease (22). A most recent study found that LPS-activated macrophages secreted Neu1 sialidase to desialylate the cell surface of target tumor cells and facilitate the programmed cell removal (PrCR) of tumor cells (23). Collectively, these findings indicate that immune cell activation causes sialidase relocation and secretion, which could cause the desialylation of glycoproteins and glycolipids of both its own cell surface and target cell surface, as well as in the extracellular environment (24). However, the molecular mechanisms related to LPS-activated sialidase expression, re-location and secretion are still incomplete.

THP-1 is a human leukemia monocytic cell line derived from an acute monocytic leukemia patient, which has been extensively used to study monocyte/macrophage functions, mechanisms, and signaling pathways (25–27). In this study, we systematically profiled the desialylation of THP-1 monocytes and THP-1 macrophages upon LPS activation. Specifically, we examined total Sia level changes (desialylation), sialidases such as Neu1 and Neu3 activity, expression, and secretion from THP-1 monocytes and THP-1 macrophages upon LPS activation. These research results contribute to discovering molecular desialylation mechanisms in controlling the LPS/TLR4 signaling pathway in monocytes and macrophages, which could impact the development of novel therapeutic approaches for modulating deregulated LPS/TLR4 signaling pathways such as in sepsis disease (28) and activated macrophage-mediated programmed cell removal (PrCR) anticancer therapy (29).

## Materials and methods

### Reagents

PMA (phorbol-12-myristate-13-acetate), DANA (2,3-dehydro-2-deoxy-*N*-acetylneuraminic acid), and LPS from *Escherichia coli* (L8274-25MG) were purchased from Sigma (St. Louis, Missouri). The Macrophage Marker Antibody Panel (CD11b, CD68, CD163, CD14, CD16) was acquired from Abcam (ab254013, Lot GR3441283-2, Cambridge, UK). *N*-Acetyl-D-neuraminic acid-1,2,3-^13^C_3_ (^13^C_3_-Sia, 99%) were obtained from the Carbosynth US LLC. (San Diego, CA). Acetonitrile (HPLC grade), paraformaldehyde (PFA, 16% w/v), and acetic acid (ACS grade) were supplied by Fisher Scientific (Hanover Park, IL, USA). FITC-labeled Arachis hypogaea (peanut) (PNA) lectin and PNA-biotin were obtained from bioWORLD (Dublin, OH). 4-Methylumbelliferyl *N*-acetyl-α-D-neuraminic acid (4-MU-Neu5Ac) sodium salt was purchased from Carbosynth LLC (San Diego, CA, USA). GM3 ganglioside was obtained from Avanti Polar Lipids (Alabaster, AL, USA). FACS buffer (eBioscience, 00-4222-26). Human Sialidase-1 ELISA kit (#MBS7269288), Neu1 polyclonal antibody (Product # PA5-42552) and Human Sialidase-3 ELISA Kit (#MBS1662490 were from MyBiSource (CA, USA). Neu3 monoclonal antibody (Ab) was from MBL International (Woburn, MA). β-Actin (C4) mouse monoclonal IgG antibody was from Santa Cruz Biotechnology (Dallas, TX). Goat anti-Mouse IgG (H+L) was from Invitrogen (Carlsbad, CA). streptavidin-HRP was from Vector Laboratories.

### Apparatus

LC-MS/MS quantification was performed using a Nexera liquid chromatography system (Shimadzu, Columbia, MD, USA) coupled with a Qtrap 5500 mass spectrometer equipped with an electrospray ionization source (AB SCIEX, Framingham, MA, USA), and the data were analyzed using Analysis 1.6.1 software. A FACSCanto II system was used for the flow cytometry and data was assessed by the BD FACSDiva software (BD Bioscience, Mountain View, CA). Tomy MX-305 High-Speed Micro Centrifuge (BioSurplus, Inc. CA. USA). ELISA absorbance spectra measured using a SpectraMax iD3 Multi‐Mode Microplate Reader (Molecular Devices). Automated cell counter (Life Technologies, Carlsbad, CA, # A49891).

### Cell culture

THP-1 monocytes (ATCC^®^ TIB-202™) obtained from ATCC were cultured in RPMI 1640 medium supplemented with 10% fetal bovine serum (FBS; Gibco, Rockville, MD) and 1% penicillin/streptomycin (P/S; Gibco, Rockville, MD) at 37 °C with 5% CO_2_. THP-1 macrophages were differentiated from THP-1 monocytes by treating with PMA (10 ng/mL) for 48 h in RPMI 1640 medium containing 10% heat-inactive FBS and 1% P/S 37 °C with 5% CO_2_. In addition, THP-1 macrophages were treated with 100 ng/mL LPS alone for 24 h (constituted in 1× phosphate-buffered saline (PBS)). Cells were washed twice with ice-cold PBS and lysed with RIPA buffer on ice for 5 minutes. The cell lysates were collected and clarified by centrifugation at 15,000 g at 4 °C for 10 minutes. Protein concentrations were measured using the Bradford method with a protein assay kit (Bio-Rad, Hercules, CA, USA).

### Enzyme activity

The cell lysates were resuspended in the reaction buffer containing 0.05 M sodium acetate at pH 4.5, 0.1% Triton X-100, and 0.1% BSA or pH 7.4, PBS. The activity of Neu1 and Neu3 were determined by the addition of 0.125 mM 4-MU-Neu5Ac and 0.250 mM ganglioside GM3 with or without 10 µM sialidase inhibitor 2,3-dehydro-2-deoxy-N-acetylneuraminic acid (DANA), respectively. The cell culture medium was collected from monocytes and macrophages with or without LPS stimulation. The activity of Neu1 and Neu3 in the cell culture medium was determined by mixing 10 μL sample into 0.05 M sodium acetate at pH 4.5 or 0.1% Triton X-100, and 0.1% BSA of PBS at pH 7.4, which contains 0.125 mM 4-MU-Neu5Ac and 0.250 mM ganglioside GM3 with or without 10 µM sialidase inhibitor DANA, respectively (Lajaunias et al., 2005; Feng et al., 2013; Sanderson et al., 2019). After 0.5 h incubation at 37 °C, the reaction mixtures were centrifuged at 2500rpm to remove cellular debris, and the supernatants were precipitated with acetonitrile at a 1:3 ratio to remove the soluble proteins. Then 20 μL of the supernatant was mixed with 5 μL of internal standard (^13^C_3_-Sia) and 75 μL of PBS (pH7.4), and 5 μL of the mixture was subjected to the LC-MS/MS analysis. Protein concentrations in each assay were measured by the Bradford method using a protein assay kit (Bio-Rad, Hercules, CA, USA). One unit of sialidase activity was defined as 1 nmol of free Sia that was released from 1 mg of total protein per hour at 37 °C.

### LC-MS/MS sample preparation

To assess the cellular Sia content, a sample containing 1,000,000 cells/mL was collected and homogenized using ultrasonication. The cell lysis was then hydrolyzed with 2 M acetic acid (1:1) at 80 °C for 90 minutes. 5 μL of a 500 ng/mL internal standard (IS) solution (^13^C3-Neu5Ac) was subsequently added to 95 μL of the resulting lysate. The combination was then injected into an LC-MS/MS analytical apparatus.

### LC-MS/MS analysis

The Primesep D column (2.1 × 100 mm, 5 μm; SIELC Technologies, Prospect Heights, IL, USA) was used to separate the Neu5Ac and IS (^13^C_3_-Neu5Ac). The binary gradient was used in the HPLC, in which phase A was deionized water with 10 mM ammonium formate and 0.1% formic acid, and phase B was 90% acetonitrile and 10% water with 3 mM ammonium formate and 0.04% formic acid. The column was balanced and started with 10% phase B, which lasted for 1 min, then the gradient was changed to 95% B within 3 min and kept at 95% B for 2 min. Finally, the mobile phase was changed to 10% B in 0.1 min and maintained for 2.9 min. For each run of the samples, 9 min were required. To qualitatively measure the amount of Sia, the MRM transitions were set at *m/z* 308→87 for Neu5Ac and 311→ 90 for IS ^13^C-Neu5Ac.

### Method validation and sample preparation

Sia calibration standard stock solution was prepared in PBS (pH 7.4) at concentrations of 20, 50, 150, 500, 1500, 4500, 10000 ng/mL. Quality control (QC) samples of stock solutions were prepared at low (40 ng/mL), mid (800 ng/mL), and high (8000 ng/mL) concentrations under the same conditions. Internal standard (^13^C_3_-Sia) stock solution was prepared in PBS at 500 ng/mL. To prepare the sample, IS stock solution (5 µL), standard or QC stock solution (5 µL), and mobile phase A (90 µL) were mixed well, and 5 µL of the mixture was injected into the LC-MS/MS for analyzing.

### Flow cytometry

Flow cytometry was used to determine the cell surface Gal; THP-1 monocytes and THP-1 derived macrophages (100,000 cells) were treated with LPS (100 ng/mL) for 24 h. The treated cells were collected utilizing mechanically detachment pipette and washed with PBS and stained with PNA-FITC (20 µg/mL) at room temperature for 30 min. Cells were subsequently washed to remove unbound PNA-FITC, re-suspended in cold PBS and determined by a FACSCantoII flow cytometer operated through BD FACSDiva software (BD Bioscience, Mountain View, CA, USA) and analyzed using FlowJo software.

### Western blot

Cells in the indicated conditions were lysed with a mixture of cold RIPA buffer and protease inhibitor cocktail (1:100, v/v) on ice for 5 min, and the cell lysates were collected and clarified by centrifugation at 15,000 x*g* at 4°C for 10 min. Protein concentrations were measured by Bradford method using a protein assay kit. Lysates (30 μg/sample) were then run on sodium dodecyl sulfate-polyacrylamide gel electrophoresis using a 4 to 12% bis-acrylamide gel at 110 V for 2 h. The gel was transferred to nitrocellulose using a Trans-Blot Turbo transfer buffer at 20 V for 20 min. The blot was blocked with 5% free fat milk in Tris-buffered saline and incubated with specific primary antibodies against Neu1 and Neu3 (1:1,000 dilutions) overnight at 4 °C, followed by goat anti-mouse IgG or anti-rabbit IgG (1:2,000 dilutions) at room temperature for 1 h with a dilution of 1:3000 in blocking buffer. After exposing the membrane to the ECL reagent, it was exposed to x-ray film to detect the signal.

### Lectin blot and immunoblot

Cell lysates were prepared in RIPA lysis buffer (50 mM Tris-HCL, pH 8.0, with 150 nM sodium Chloride, 1.0% lgepal CA-630 (NP-40), 0.5% sodium deoxycholate, 0.1% sodium dodecyl sulfate, including protease and phosphatase inhibitor cocktails), incubated for 20 minutes, and centrifuged at 13,000 rpm for 10 minutes and then analyzed by lectin blot. The concentration of the running gel was 10%. After blocking with 1% BSA, for lectin blot, membranes were incubated PNA-biotn (1:1,000 dilution) (1 μg/mL) followed by incubation with Streptavidin-HRP (1:10,000 dilution). For western blot of control protein β-actin, membranes were incubated with the primary antibody β-actin mouse monoclonal IgG (1:2,000 dilution) followed by incubation with secondary antibody Goat anti-Mouse IgG (H+L) (1:3,000 dilution). The signal was detected with an enhanced chemiluminescence (ECL) kit.

### ELISA

The level of Neu1 and Neu3 in the cell culture supernatant was quantified by competitive enzyme immunoassays performed according to the manufacturer’s protocol. Briefly, the cell culture was centrifuged at 3000 rpm for 15 minutes to remove debris. The capture antibody was added to flat bottom 96-well ELISA plates at 4 °C according to the manufacturer’s instructions. Then, the plate was incubated with 100 μL of standards and cell culture supernatant, followed by a specific biotinylated antibody, and incubated for 1 hour and washed three times. Then the 96-well plate was incubated with avidin peroxidase. The TMB substrate was added as substrate, and the reaction was stopped after 20 minutes with 50 μL of stop solution. Finally, the plates were scanned at 450 nm by the SpectraMax iD3 Multi‐Mode Microplate Reader (Molecular Devices).

### Statistical analysis

Unless otherwise noted, all data was represented as average ± standard error (SE) from n = 3 wells/conditions, with at least three independent repeats of each assay. Significance in differences between groups was analyzed using one-sample *t*-test, unpaired *t* test, and One-Way ANOVA with Tukey’s multiple comparisons test, respectively. * Indicates the significant differences. *p <0.05, **p <0.005, ***p < 0.001, ****p < 0.0001. The assumptions required for parametric tests were not tested.

## Results

### THP-1 monocytes differentiation to THP-1 macrophages

**Figure 1:**
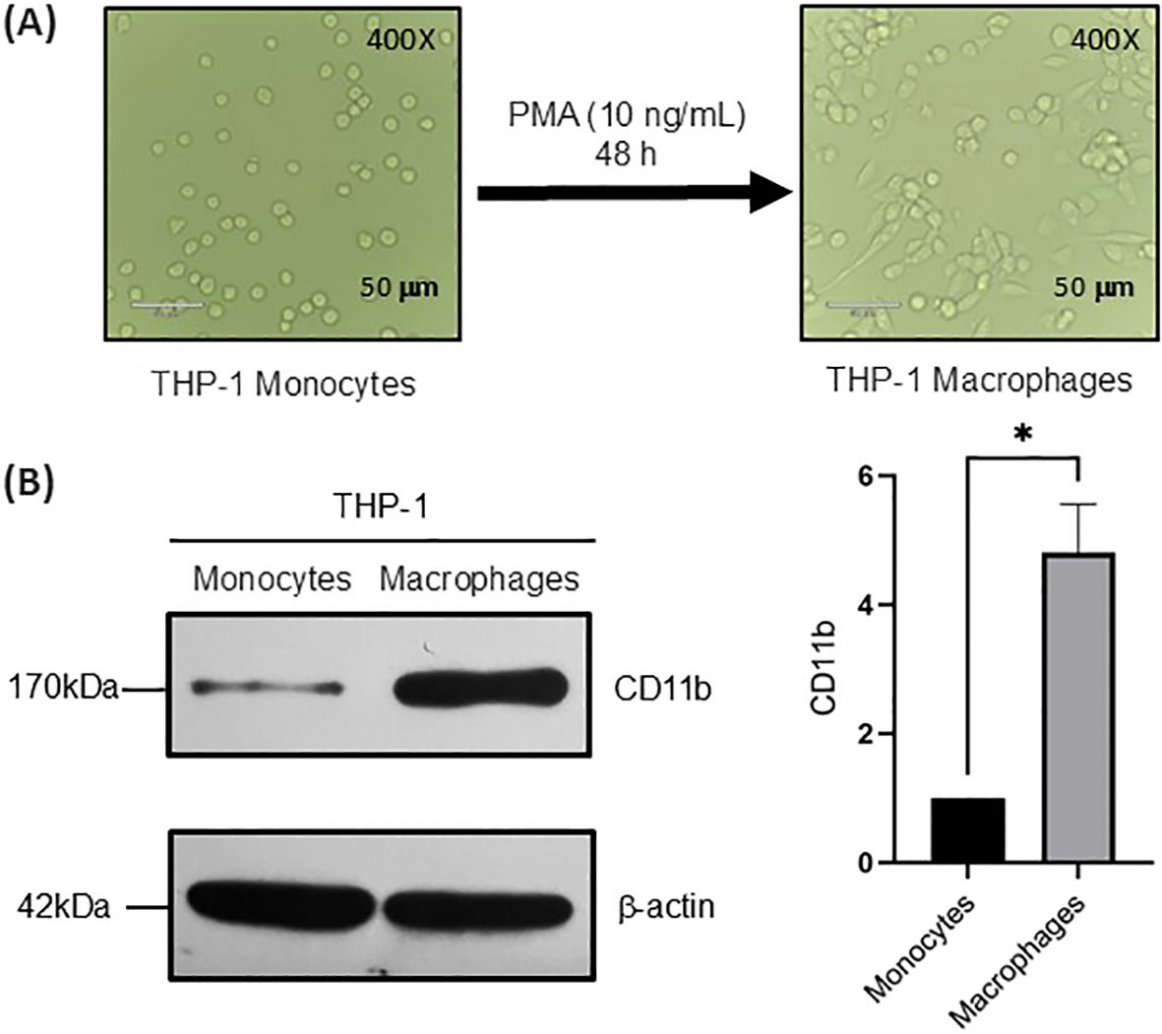
THP-1 monocytes differentiation to THP-1 macrophages. **(A)**. Morphology of THP-1 monocytes and following treatment with PMA (100 ng/mL) for 48 h. THP-1 cells adhered to the culture flask and differentiated into macrophages. Magnification, ×400. Scale bar = 50 μm. **(B)**. Western blot of macrophage biomarker CD11b. Statistical analysis was done by one-sample *t*-test. * indicates p < 0.01 between the groups. Data were presented as average ± SE (n = 3 wells/condition), with three independent repeats of the experiment.

THP-1 monocytes could differentiate to macrophages *via* phorbol-12-myristate-13-acetate (PMA) (27, 30). In this study, THP-1 monocytes were incubated with PMA at 10 ng/mL for 48 h. The differentiation of THP-1 monocytes to macrophages was confirmed by cell morphology (**Figure 1A**). THP-1 monocytes displayed a small and oval shape in non-adherent pattern, while macrophage-like THP-1 cells were larger and adherent, with round and flat shapes. In addition, the expression of CD11b showed more than 80% positive after 48 h treatment with PMA (**Figure 1B**) (30). These results indicate a successful differentiation of THP-1 monocytes to THP-1 macrophages.

### The desialylation of THP-1 monocytes and THP-1 macrophages activated by LPS

**Figure 2:**
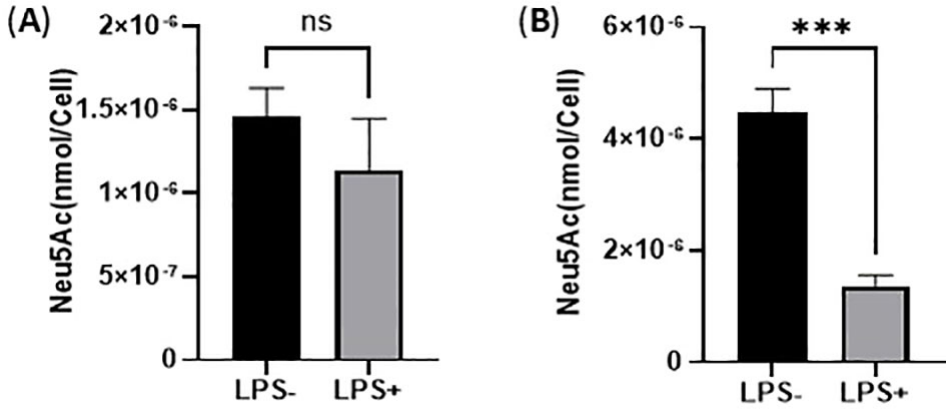
Total Sia in THP-1 monocytes and THP-1 macrophages upon LPS activation (100 ng/mL, 24 h). **(A)**. Total Sia level in cell lysates of THP-1 monocytes upon LPS activation; **(B)**. Total Sia level in cell lysates of THP-1 macrophages upon LPS activation (24 h). Statistical analysis was done by unpaired *t* test. * indicates p < 0.01 between the groups, ** indicates p < 0.005 between the groups, *** indicates p < 0.001 between the groups. Data were presented as average ± SE (n = 3 wells/condition), with three independent repeats of the experiment.

**Figure 3:**
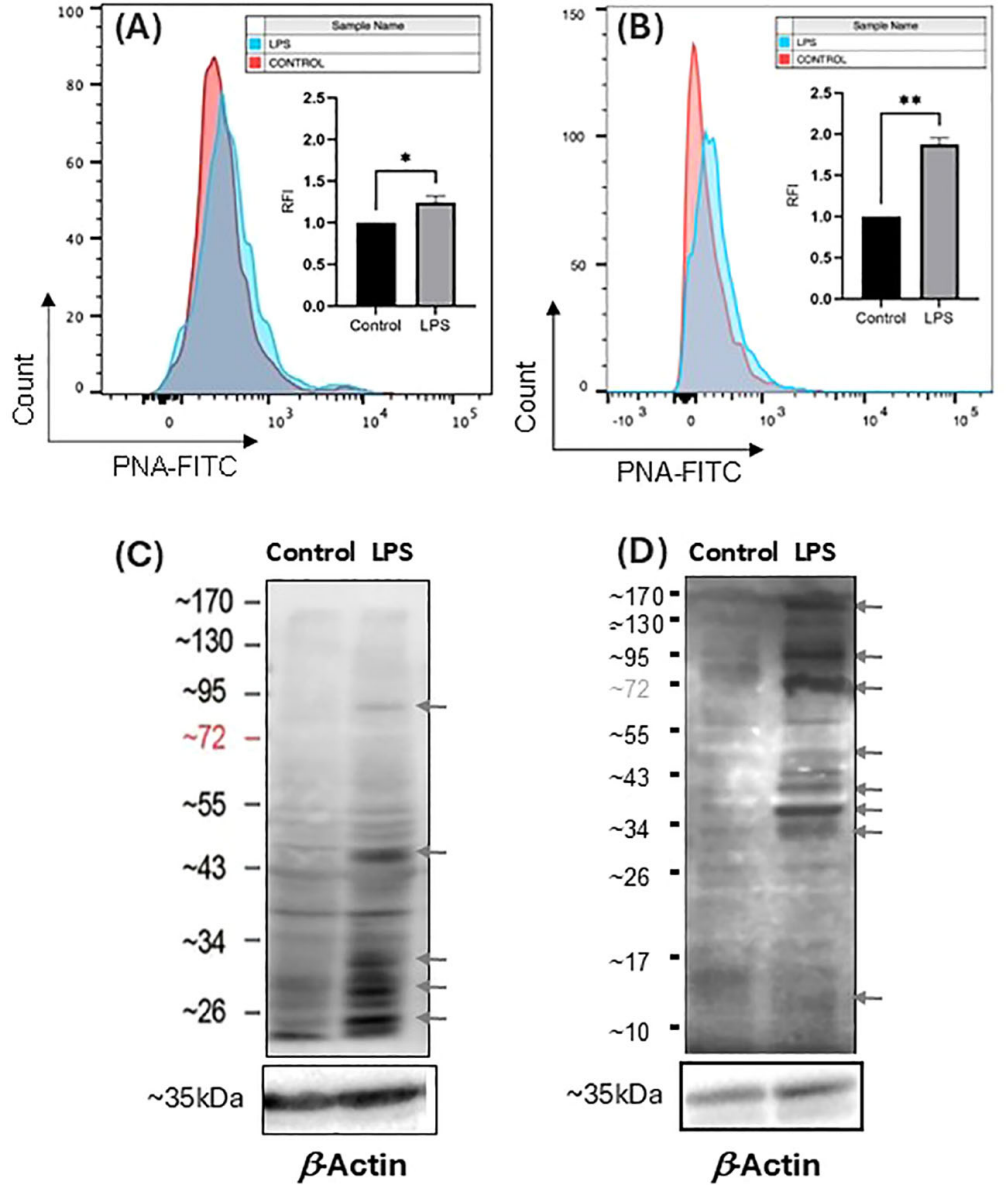
Desialylation of THP-1 monocytes and THP-1 macrophages upon LPS activation by determining Gal exposure. Flow cytometry examination of cell surface desialylation of THP-1 monocytes **(A)** and THP-1 macrophage **(B)** by PNA-FITC binding of Gal. Lectin blot examination of total desialylation of cell lysates of THP-1 monocytes **(C)** and THP-1 macrophages **(D)** by PNA-biotin binding of Gal with β-actin used as the loading control. Statistical analysis was done by one-sample *t*-test. * indicates p < 0.01 between the groups, ** indicates p < 0.005 between the groups. Data were presented as an average ± SE (n = 3 wells/condition), with three independent repeats of the experiment.

LPS activation of monocyte and macrophage could cause endogenous sialidase expression, which causes desialylation that enhances secretion of the pro-inflammatory cytokines, including TNF-α and IL-6 (31, 32). To examine the desialylation extent, the total Sia level in cell lysates of THP-1 monocytes and THP-1 macrophages were quantified with our previously developed LC-MS/MS method, which used ^13^C_3_-Neu5Ac as an internal standard (IS) to generate a liner large calibration range from 2.00 to 2.00 × 10^3^ ng/mL (y = 983.69x + 405658, R^2^ = 0.9984) for determining small amounts of free sialic acid in the cell lysates (33). Briefly, ^13^C_3_-Neu5Ac was used as an internal standard (IS). The mass detection for both Neu5Ac and ^13^C_3_-Neu5Ac was carried out in negative electrospray ionization and multiple reaction monitoring (MRM) mode. We selected transitions of m/z 308 → 87 for Neu5Ac, 311 → 91 for ^13^C_3_-Neu5Ac as the MRM channels for quantification. The transitions of 308 → 170 for Neu5Ac and 311→ 173 for ^13^C_3_-Neu5Ac were also monitored during LC-MS/MS analysis for quality assurance. As a result, the total Sia levels decreased in the cell lysates of both THP-1 monocytes and THP-1 macrophages after LPS activation (100 ng/mL, 24 h) (**Figures 2A, B**), indicating that desialylation of the total glycoconjugates of THP-1 monocyte and THP-1 macrophage occurred during LPS activation. Sias attach to galactose (Gal) or *N*-acetylgalactosamine (GalNAc) through α2,3- or α2,6-linkage (1). Desialylation could cause exposure of Gal or GalNAc on the cell surface glycoproteins. Therefore, to confirm the cell surface sialylation level change, we examined cell surface Gal exposure of THP-1 monocytes and THP-1 macrophages treated with and without LPS with FITC-labeled *Arachis hypogaea* (peanut) (PNA), which explicitly binds to the terminal β-Gal (34). As a result, an increase in PNA-FITC staining was observed, indicating that Gal level increased on the cell surface of both THP-1 monocytes and THP-1 macrophages upon LPS activation (**Figures 3A, B**). The significant increase of Gal level on the cell surface of THP-1 macrophages (**Figure 3B**) is correlated to significant desialylation of THP-1 macrophages (**Figure 2B**), suggesting that THP-1 macrophages are more highly desialylated than THP-1 monocytes upon LPS activation. Total cellular desialylation was also examined by lectin blot for the cell lysates of THP-1 monocytes and THP-1 macrophages upon LPS activation, respectively. As a result, there are apparent PNA binding for many proteins in both THP-1 monocytes (**Figure 3C**) and THP-1 macrophages (**Figure 3D**) upon LPS activation, indicating a significant desialylation. Interestingly, the desialylation occurred for different proteins in comparison between THP-1 monocytes and THP-1 macrophages. These desialylated proteins will be profiled by lectin pull down and proteomic study in the future. It should be noted that PNA specifically recognizes the Galβ1-3GalNAc structure. Other lectins detecting Gal on other glycan classes should be used for total desialylation investigation in the future.

### Enhanced sialidase activities of THP-1 monocytes and THP-1 macrophages upon LPS activation

**Figure 4:**
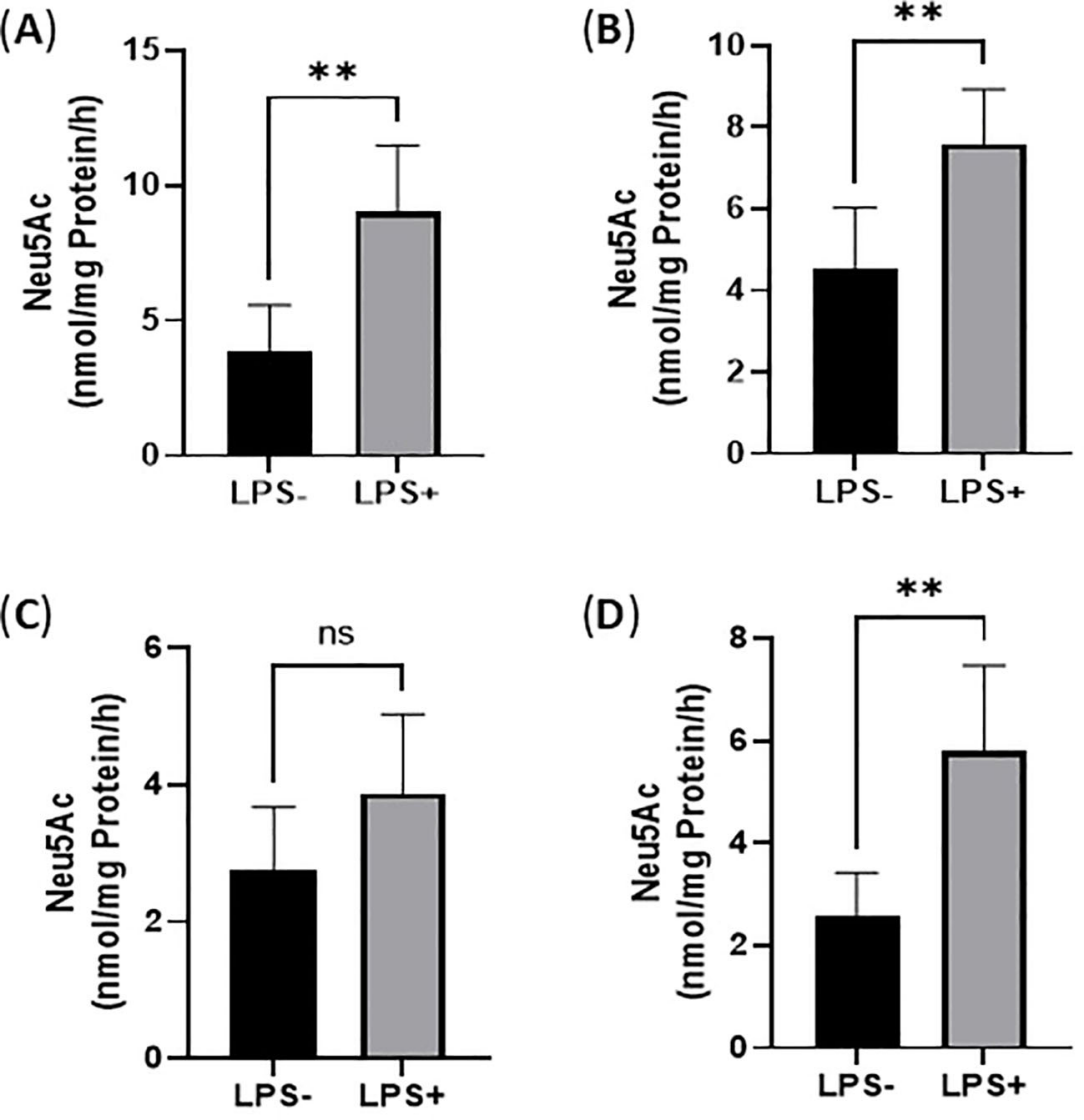
Sialidase activity of THP-1 monocytes upon LPS activation. **(A)**. Sialidase activity against 4-MU-Neu5Ac at pH 4.5 in cell lysates; **(B)**. Sialidase activity against GM3 at pH 4.5 in cell lysates; **(C)**. Sialidase against with 4-MU-Neu5Ac at pH 4.5 in cell culture medium; **(D)**. Sialidase activity against GM3 at pH 4.5 in cell culture medium. Statistical analysis was done by unpaired *t* test. * indicates p < 0.01 between the groups, ** indicates p < 0.005 between the groups. Data were presented as average ± SE (n = 3 wells/condition), with three independent repeats of the experiment.

LPS activation could cause endogenous sialidase expression that catalyzes the removal of Sia from both monocytes and macrophages. In this study, the sialidase activity of cell lysates of THP-1 monocytes and THP-1 macrophages treated with and without LPS (100 ng/mL for 24 h) were quantified by measuring the released free Sia from sialidase substrate by LC-MS/MS method. Specifically, sialidase activity against 4-MU-Neu5Ac was examined at pH 4.5 by following the literature method (35). Sialidase activity against GM3 was examined at pH 4.5 as well as described in the literature (36). As a result, sialidase activity increased 1.3-fold when examined on 4-MU-Neu5Ac (**Figure 4A**) but increased 0.68-fold when examined on GM3 (**Figure 4B**) in the cell lysates of THP-1 monocytes upon LPS activation. 4-MU-Neu5Ac and ganglioside GM3 are often used as substrates for Neu1 and Neu3, respectively (37). These results indicate that THP-1 monocytes highly expressed both Neu1 and Neu3 sialidase upon LPS activation.

**Figure 5:**
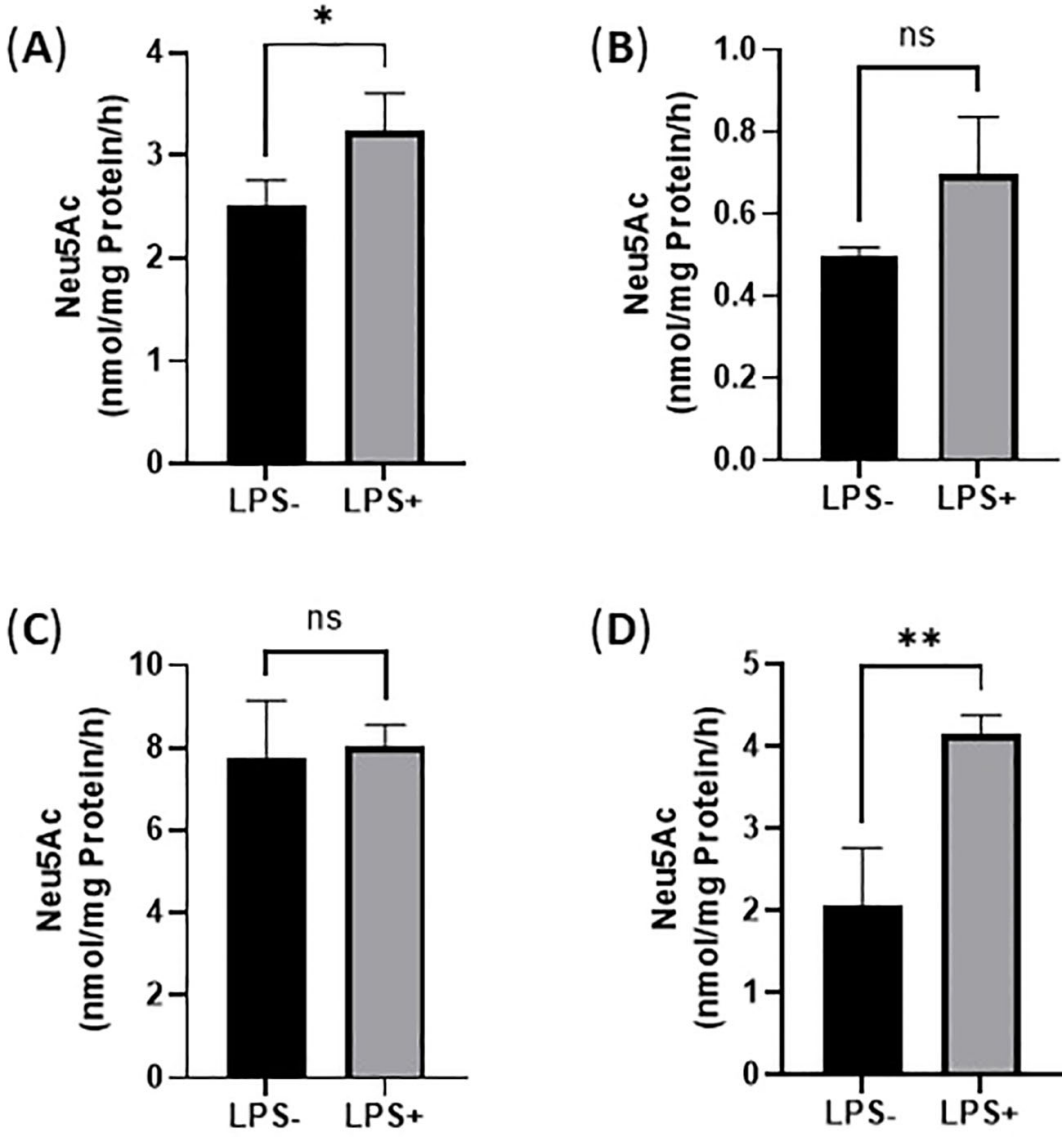
Sialidase activity in THP-1 macrophages upon LPS activation. **(A)**. Sialidase activity against 4-MU-Neu5Ac at pH 4.5 in cell lysates; **(B)**. Sialidase activity against GM3 at pH 4.5 in cell lysates; **(C)**. Sialidase activity against 4-MU-Neu5Ac at pH 4.5 in cell culture medium; **(D)**. Sialidase activity against GM3 at pH 4.5 in cell culture medium. Statistical analysis was done by unpaired *t* test. * indicates p < 0.01 between the groups, ** indicates p < 0.005 between the groups. Data were presented as average ± SE (n = 3 wells/condition), with three independent repeats of the experiment.

Next, we quantified Neu1 and Neu3 sialidase activity of cell lysates of THP-1 macrophages treated with and without LPS (100 ng/mL for 24 h) with the exogenous sialidase substrates 4‐MU‐Neu5Ac and GM3 at pH 4.5, respectively. As a result, sialidase activity increased 1.3-fold when examined on 4-MU-Neu5Ac (**Figure 5A**) but increased 1.4-fold when examined on GM3 (**Figure 5B**) in cell lysates of THP-1 macrophages upon LPS activation. These results indicate that THP-1 macrophages highly expressed both Neu1 and Neu3 sialidase upon LPS activation.

### Sialidase expression levels of THP-1 monocytes and THP-1 macrophages upon LPS activation

**Figure 6:**
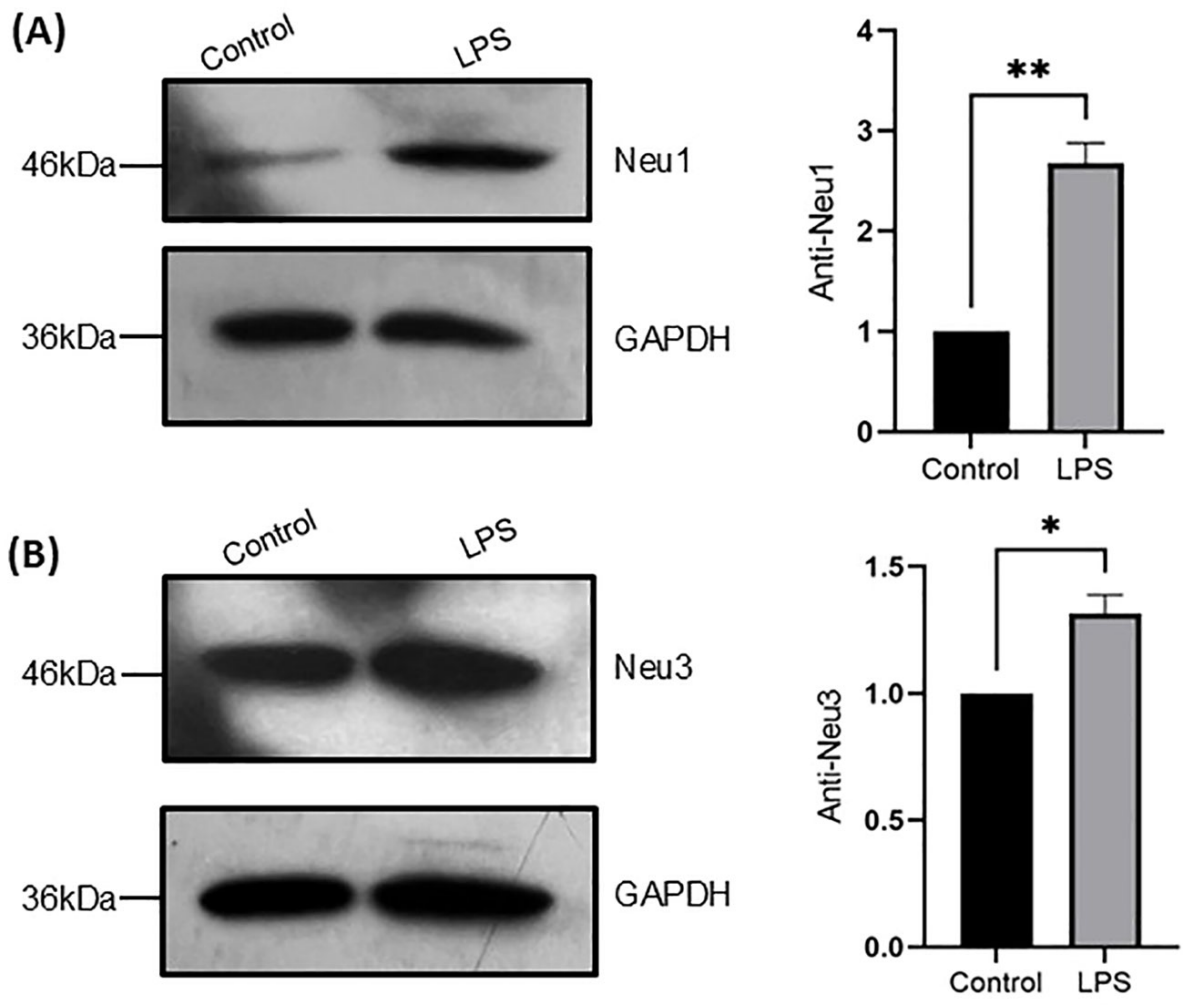
Sialidase levels in THP-1 monocytes upon LPS activation. **(A)** Neu1 protein expression of THP-1 monocyte cells without and with LPS treatment; **(B)** Neu3 protein expression of THP-1 monocyte cells without and with LPS treatment. Neu1 and Neu3 expressions were normalized to GAPDH expression in the same lane. Statistical analysis was done by one-sample *t*-test. * indicates p < 0.01 between the groups, ** indicates p < 0.005 between the groups. Data were presented as average ± SE (n = 3 wells/condition), with three independent repeats of the experiment.

**Figure 7:**
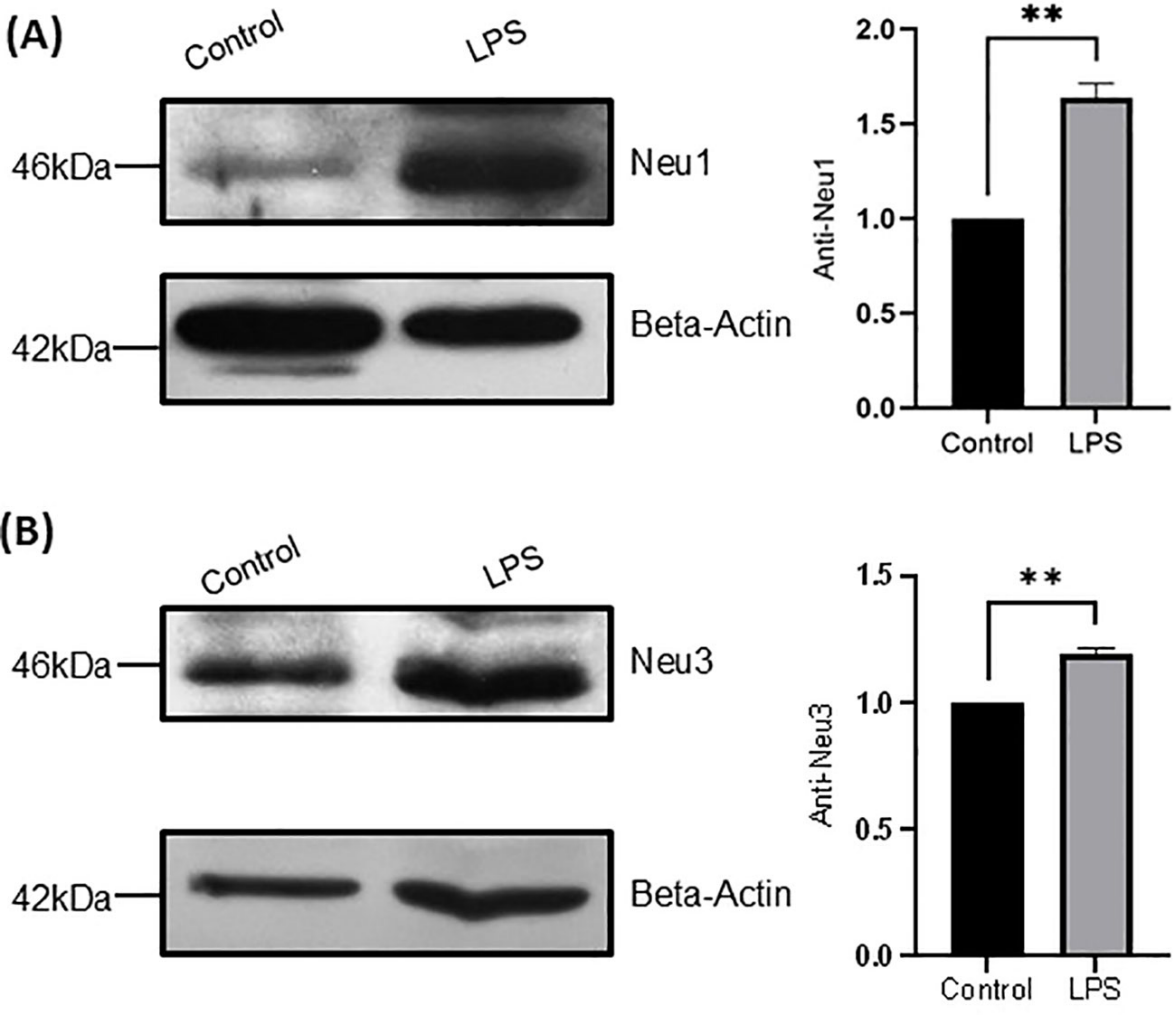
Sialidase levels in THP-1 macrophages upon LPS stimulation. **(A)**. Neu1 protein expression of THP-1 macrophage cells without and with LPS treatment; **(B)** Neu3 protein expression of THP-1 macrophage cells without and with LPS treatment. Neu1 and Neu3 expressions were normalized to beta actin expression in the same lane. Statistical analysis was done by one-sample t-test. * indicates p < 0.01 between the groups, ** indicates p < 0.005 between the groups. Data were presented as average ± SE (n = 3 wells/condition), with three independent repeats of the experiment.

As we found that increased sialidase activities of THP-1 monocytes upon LPS activation, we further examined the expression levels of sialidases in THP-1 monocytes treated with and without LPS (100 ng/mL for 24 h) by western blot. As a result, Neu1 and Neu3 sialidase expressions were detected but not cytosolic Neu2 and Neu4 sialidase and both Neu1 and Neu3 increased significantly in THP-1 monocytes upon LPS activation compared with the control (**Figures 6A, B**). This is consistent with the increased sialidase activity measured with the substrate 4-MU-Neu5Ac and GM3 above (**Figure 4**). Next, we examined the expression of Neu1 and Neu3 sialidase in THP-1 macrophages treated with and without LPS (100 ng/mL for 24 h) as well. As a result, both Neu1 and Neu3 expression also increased significantly in cell lysates of THP-1 macrophages upon LPS activation compared with the control (**Figures 7A, B**). This is consistent with the increased sialidase activity measured with the substrate 4-MU-Neu5Ac and GM3 in cell lysates of THP-1 macrophages upon LPS activation as well (**Figure 5**). All these results confirmed the increased expression of both Neu1 and Neu3 in both THP-1 monocytes and THP-1 macrophages upon LPS activation.

### Sialidase secretion from THP-1 monocytes and THP-1 macrophages upon LPS activation

Next, we quantified sialidase activity in spent media/conditioned media collected from THP-1 monocytes and THP-1 macrophages treated with and without LPS (100 ng/mL for 24 h). As a result, sialidase activity increased 1.4-fold when examined on 4-MU-Neu5Ac (**Figure 4C**) but increased 2.3-fold when examined on GM3 (**Figure 4D**) in the spent media/conditioned media collected from THP-1 monocytes activated with LPS. However, the sialidase activity increased 1.04-fold when examined on 4-MU-Neu5Ac (**Figure 5C**) but increased 2.0-fold when examined on GM3 (**Figure 5D**) in the spent media/conditioned media collected from THP-1 macrophages activated with LPS. Overall, macrophages have less sialidase activity against 4-MU-Neu5Ac in cell lysates without treatment with LPS (**Figures 5A, B**) compared to monocytes (**Figures 4A, B**), which indicates that more sialidases are released from macrophages even under the control condition. This is supported by the result above that cell culture medium from macrophages showed much more sialidase activity (**Figures 5C, D**) compared to monocytes (**Figures 4C, D**).

**Figure 8:**
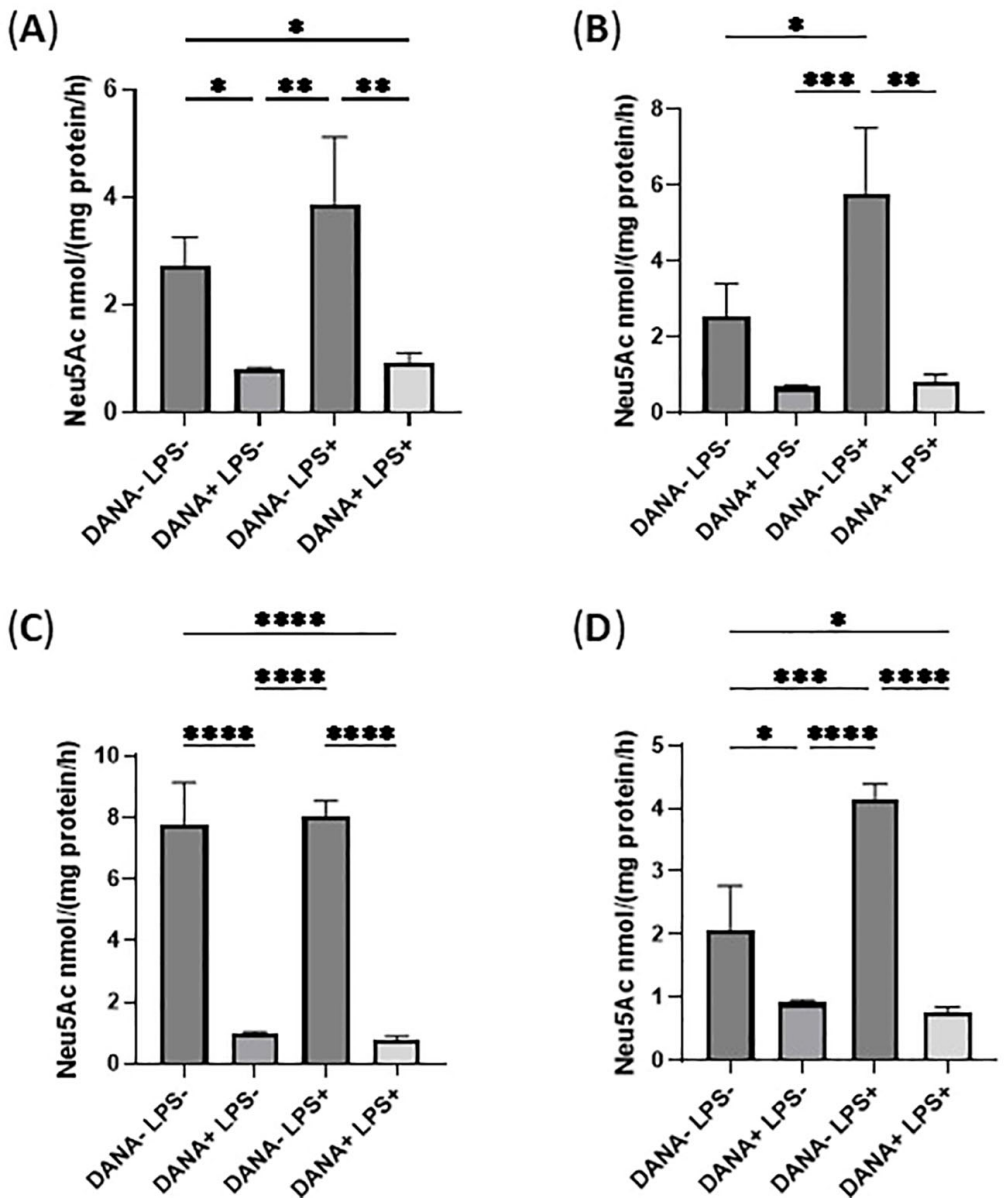
Sialidase activity in THP-1 monocyte and THP-1 macrophage cell culture medium upon LPS activation. **(A)**. Sialidase activity against 4-MU-Neu5Ac at pH4.5 in THP-1 monocytes cell culture medium with or without sialidase inhibitor DANA; **(B)**. Sialidase activity against GM3 at pH4.5 in THP-1 monocytes cell culture medium with or without sialidase inhibitor DANA; **(C)**. Sialidase activity against 4-MU-Neu5Ac at pH 4.5 in THP-1 macrophage cell culture medium with or without sialidase inhibitor DANA; **(D)**. Sialidase activity against GM3 at pH4.5 in THP-1 macrophage cell culture medium with or without the sialidase inhibitor DANA. Statistical analysis was done by One-way ANOVA with Tukey’s multiple comparisons test. * indicates p < 0.05 between the groups, ** indicates p < 0.005 between the groups, *** indicates p < 0.001, **** indicates p < 0.0001 between the groups. Data were presented as average ± SE (n = 3 wells/condition), with three independent repeats of the experiment.

Multiple enzymes exist in the cell culture medium, which may also cause hydrolysis of the sialidase substrate 4-MU-Neu5Ac and GM3 (38). In this study, the sialidase inhibitor DANA was used to confirm the specific sialidase activity in the cell culture media. As a result, DANA inhibited sialidase activities against both 4-MU-Neu5Ac and GM3 in the cell culture medium of THP-1 monocytes (**Figures 8A, B**) and THP-1 macrophages (**Figures 8C, D**). These results indicate that the sialidases that exist in the culture media are secreted from THP-1 monocytes and THP-1 macrophages upon LPS activation.

**Figure 9:**
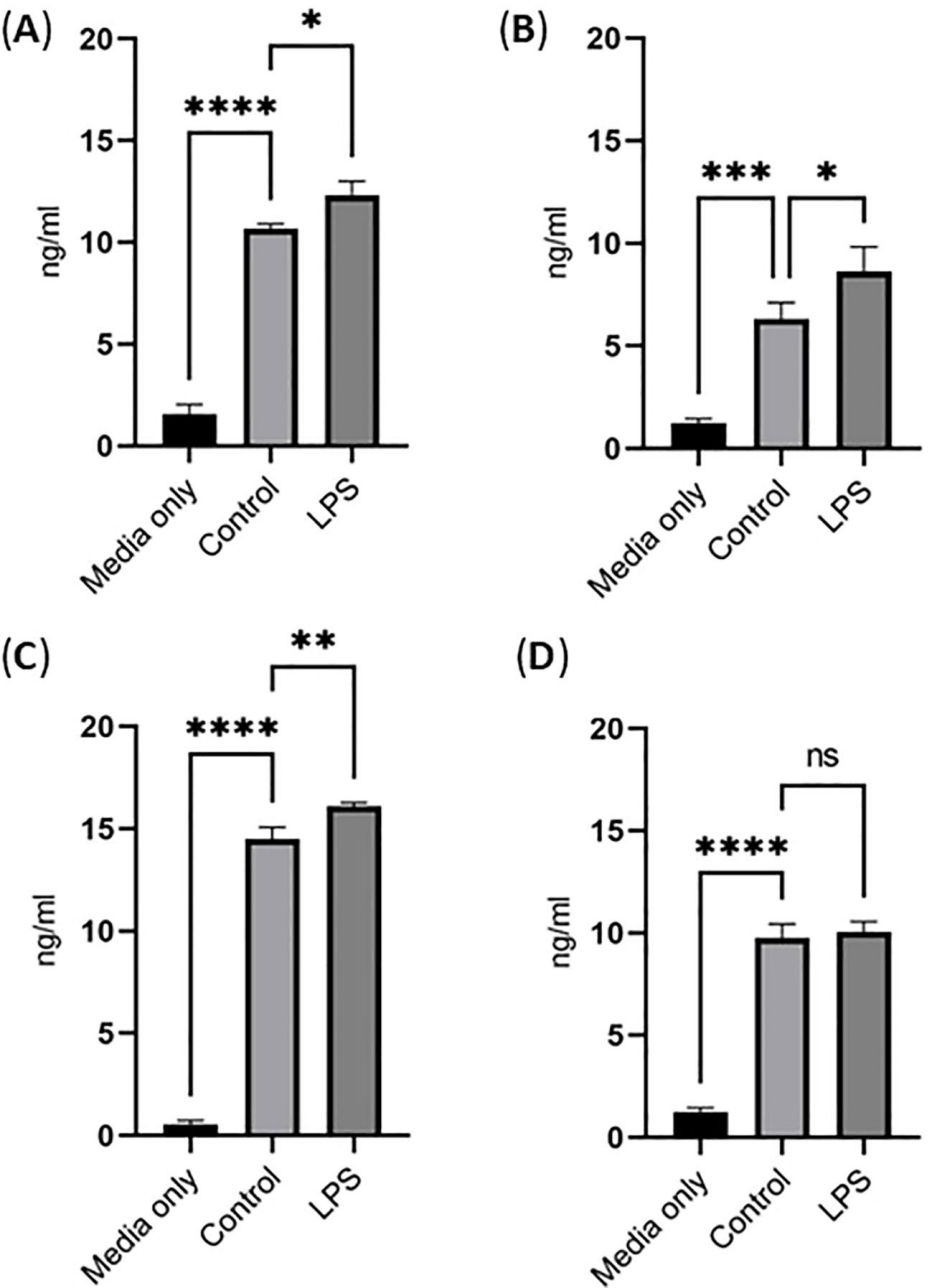
Sialidase levels in the cell culture media of THP-1 monocytes and THP-1 macrophages upon LPS activation. ELISA quantification of Neu1 **(A)** and Neu3 **(B)** released in the supernatants of THP- monocytes and Neu1 **(C)** and Neu3 **(D)** released in the supernatants of THP- macrophages upon LPS stimulation. Statistical analysis was done by One-way ANOVA with Tukey’s multiple comparisons test. * indicates the significant differences. *p < 0.05, **p < 0.005, ***p < 0.001, ****p < 0.0001. Data were presented as average ± SE (n = 3 wells/condition), with three independent repeats of the experiment.

Finally, the sialidase levels in the cell culture media of THP-1 monocytes and THP-1 macrophages with and without LPS activation (100 ng/mL for 24 h) were examined by ELISA. Surprisingly, there was some amount of both Neu1 and Neu3 in the medium that contains 10% fetal bovine serum (FBS), even in the absence of cells. However, there were much more Neu1 and Neu3 in the cell culture media in the presence of THP-1 monocytes and THP-1 macrophages under both control conditions and LPS activation (**Figure 9**). Particularly, Neu1 levels increased significantly in the cell culture media of both THP-1 monocytes (**Figure 9A**) and THP-1 macrophages upon LPS activation (**Figure 9C**). For Neu3, it increased in the cell culture media of THP-1 monocytes upon LPS activation (**Figure 9B**) but did not change significantly in THP-1 macrophages upon LPS activation (**Figure 9D**). Overall, these results confirmed sialidase Neu1 and Neu3 in the cell culture media, indicating that both THP-1 monocytes and THP-1 macrophages secrete sialidases in the control condition but secrete more upon LPS activation.

## Discussion

Monocytes and macrophages are members of the mononuclear phagocytes system, which are an essential component of innate immunity (39). LPS could activate monocytes and macrophages and induce subsequent NF-κB activation, and pro-inflammatory cytokine production (16). It was reported that LPS induces sialidase expression in macrophages, which could cause desialylation of cell surface TLR4 and its activation (18–20). However, most research was conducted with model cells such as HEK293T cells transfected with TLR4 (20) and the molecular mechanisms related to LPS-activated sialidase expression and the desialylation in the LPS/TLR4 signaling pathway are still incomplete. In this study, we systematically profiled the desialylation of THP-1 monocytes and THP-1 macrophages upon LPS activation. Specifically, we examined total cellular Sia level changes, sialidase activity, sialidase expression level, and sialidase secretion from THP-1 monocytes and THP-1 macrophages upon LPS activation.

Upon LPS activation, both THP-1 monocytes and THP-1 macrophages showed significant reduction of total Sias (desialylation) in the cell lysates (**Figure 2**). Glycan chains typically end with Sias, which are often linked to Gal. Desialylation leads to the exposure of Gal on the cell surface glycans. The cell surface Gal level of THP-1 monocytes and THP-1 macrophages were examined by flow cytometry by using lectin PNA-FITC, which explicitly binds to the terminal β-Gal. It was found that Gal level increased on the cell surface of both THP-1 monocytes (**Figure 3A**) and THP-1 macrophages upon LPS activation (**Figure 3B**), indicating desialylation for both cells. Cellular desialylation was also confirmed by lectin blot for the cell lysates of both THP-1 monocytes (**Figure 3C**) and THP-1 macrophages upon LPS activation (**Figure 3D**). Multiple reasons could cause the desialylation of cells. First, the enhanced sialidase expression could cause the Sia removal from glycoconjugates. Second, activated monocytes and macrophages could have different metabolic and biosynthetic rates of sialoglycoconjugates (40), which would cause the synthesis, secretion, re-taking, or recycling of Sia differently. Sialidases are the enzymes that could cause desialylation on the sialoglycoconjugates, and the expression level of sialidases is highly related to cell activation (41, 42). We examined the sialidase activity in THP-1 monocytes and THP-1 macrophage cell lysates and their cell culture media (**Figures 4** and **5**). Sialidase activities were enhanced in both the cell lysates and cell culture media of both THP-1 monocytes and THP-1 macrophage with the LPS activation, which indicates the upregulation and secretion of sialidases during LPS activation. To identify the specific sialidase expression and release, we examined Neu1 and Neu3 sialidase expression of cell lysates of THP-1 monocytes treated with and without LPS activation with western blot. We found that both Neu1 and Neu3 expressions increased significantly in cell lysates of both THP-1 monocytes and THP-1 macrophages upon LPS activation compared with the control. Finally, the Neu1 and Neu3 level in the cell culture media of both THP-1 monocytes and THP-1 macrophages upon LPS activation were confirmed by ELISA assay, which further indicates the secretion of both Neu1 and Neu3 sialidase from both THP-1 monocytes and THP-1 macrophages upon LPS activation (**Figure 9**). The observed extracellular activity of Neu1 and Neu3 sialidase is consistent with active secretion but does not demonstrate it. Passive secretion mechanisms are also possible. A full investigation of the sialidase secretion mechanisms is highly deserved as secreted sialidases could serve as exogenous sialidase to desialylate both extracellular glycoconjugates and cell surface receptors.

In conclusion, the desialylation profile of THP-1 monocytes and THP-1 macrophages upon LPS activation were systematically examined. We confirmed that LPS induces desialylation from THP-1 monocytes and THP-1 macrophages that are closely related to the enhanced sialidase Neu1 and Neu3 expression and activity. In addition, we found enhanced Neu1 and Neu3 sialidase activities and protein levels in the cell culture medium, of THP-1 monocytes and THP-1 macrophages upon LPS activation. This is the first report demonstrating the activity, expression and secretion of Neu1 and Neu3 sialidase from monocytes and macrophages upon LPS activation. These results indicate that enhanced activity and expression and secretion of Neu1 and Neu3 sialidase are involved in LPS activation pathway in monocytes and macrophages. It will be important to investigate TLR4 expression and desialyaltion, downstream signaling (NF-κB), and cytokine production in monocytes and macrophages, which would fully support previous findings that sialidases are involved in LPS/TLR4 signaling in immune and inflammation disorders.

## Data Availability

The original contributions presented in the study are publicly available. This data can be found here: https://figshare.com/articles/dataset/_b_Raw_Data_of_LC-MS-MS_Data_for_Manuscript_ID-1750968_b_/30767588?file=60048125.
